# Adsorption of Helium on Small Cationic PAHs: Influence of Hydrocarbon Structure on the Microsolvation Pattern

**DOI:** 10.1021/acs.jpca.1c05150

**Published:** 2021-08-26

**Authors:** Arne Schiller, Miriam Meyer, Paul Martini, Fabio Zappa, Serge A. Krasnokutski, Florent Calvo, Paul Scheier

**Affiliations:** † Institut für Ionenphysik und Angewandte Physik, Universität Innsbruck, Technikerstr. 25, A-6020 Innsbruck, Austria; ‡ Laboratory Astrophysics Group of the MPI for Astronomy at the University of Jena, Helmholtzweg 3, D-07743 Jena, Germany; § CNRS, LiPhy, Univ. Grenoble Alpes, F-38000 Grenoble, France

## Abstract

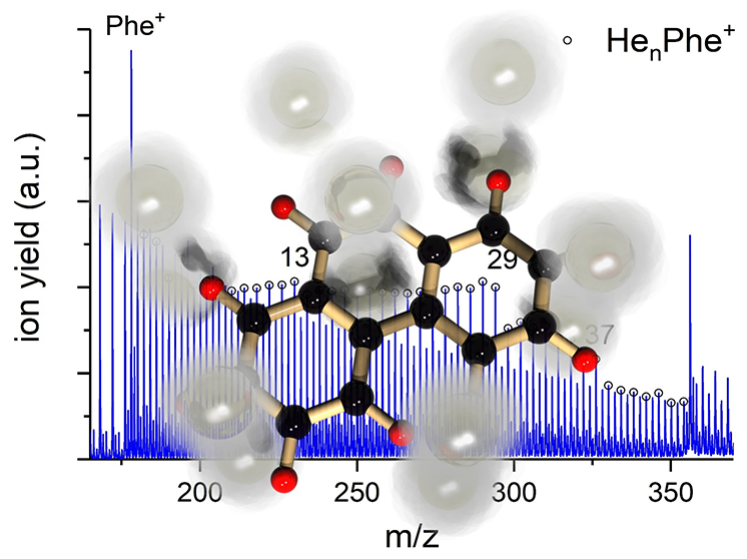

The adsorption of up to ∼100 helium atoms on cations of the planar polycyclic aromatic hydrocarbons (PAHs) anthracene, phenanthrene, fluoranthene, and pyrene was studied by combining helium nanodroplet mass spectrometry with classical and quantum computational methods. Recorded time-of-flight mass spectra reveal a unique set of structural features in the ion abundance as a function of the number of attached helium atoms for each of the investigated PAHs. Path-integral molecular dynamics simulations were used with a polarizable potential to determine the underlying adsorption patterns of helium around the studied PAH cations and in good general agreement with the experimental data. The calculated structures of the helium–PAH complexes indicate that the arrangement of adsorbed helium atoms is highly sensitive toward the structure of the solvated PAH cation. Closures of the first solvation shell around the studied PAH cations are suggested to lie between 29 and 37 adsorbed helium atoms depending on the specific PAH cation. Helium atoms are found to preferentially adsorb on these PAHs following the  commensurate pattern common for graphitic surfaces, in contrast to larger carbonaceous molecules like corannulene, coronene, and fullerenes that exhibit a 1 × 1 commensurate phase.

## 1 Introduction

The adsorption of atoms and molecules on carbonaceous materials has been widely studied for a variety of reasons, ranging from probing fundamental chemistry and physics^1−4^ to practical applications like hydrogen storage.^5−7^ Helium is a highly interesting adsorbant species from a fundamental standpoint due to its extremely weak binding and its quantum nature, which becomes relevant at the low temperatures required to bind helium atoms.^8^ Although helium is the second most abundant element in the universe by a large margin, complex formation with neutral atoms or molecules is expected to be unlikely due to its low binding energy even at the low temperatures present in most regions of the interstellar medium. The interaction of helium with ions is much stronger and, besides physisorption, makes chemisorption also possible. In fact, the helium hydride ion is considered to be the first molecule being formed in relevant quantities in the early universe.^9^ In laboratory experiments, He-tagged ions represent ideal targets for action spectroscopy, as recently demonstrated in several laboratories.^10−21^ The weak binding energy leads to small line shifts with respect to the isolated ions and is at the same time a confirmation for vibrationally cold ions.

Helium adsorption has been studied extensively on graphite,^22−26^ graphene,^27,28^ and graphene derivatives^29−32^ as well as carbon nanotubes.^33,34^ Going down in size, molecules such as fullerenes and polycyclic aromatic hydrocarbons (PAHs) offer the possibility to study helium adsorption on finite planar and curved graphene-like flakes. Whereas helium adsorption on neutral fullerenes has only been studied theoretically thus far,^35−43^ helium adsorption on neutral PAHs has been studied by using various spectroscopic and theoretical methods.^44−49^

As already mentioned, the interaction between helium atoms and ions is much stronger compared to the neutral–helium interactions due to the presence of a net charge and the resulting ion-induced polarization forces. This interaction is strongest for helium atoms closest to the ion, leading to a higher helium density in its immediate vicinity. For ions embedded in liquid helium, the helium atoms in the innermost solvation shell around the ion form a structure known as a snowball, with strongly localized helium atoms that no longer exhibit liquid, but rather solid-like character.^50^ Helium-solvated ions can be studied mass spectrometrically by monitoring their yield as a function of the number of attached helium atoms. Whereas pronounced stepwise drops in the ion yield at specific numbers of attached He atoms indicate shell closures, local intensity maxima are often designated as magic numbers, indicating particularly stable structures. Atomistic simulations can then help to elucidate the nature of the solvation structures, provided that nuclear delocalization effects are accounted for. A combined study by Leidlmair et al. utilized mass spectrometry and quantum-corrected molecular dynamics (MD) simulations of fullerene-doped helium nanodroplets (HNDs) to study the adsorption of helium on cationic fullerenes^51^ (for a review see refs (52) and (8)). The authors identified the formation of the highly ordered, rigid-like 1 × 1 commensurate phase where each hexagonal and pentagonal face is occupied by one helium atom for C_60_He_32_^+^ and C_70_He_37_^+^ as well as, tentatively, the closure of the first helium solvation shell at C_60_He_60_^+^ and C_70_He_62_^+^. While the agreement between experiment and theory was excellent for *n* = 32, the evidence was inconclusive for the second anomaly, as the MD simulations suggested the closure of the first solvation shell of C_60_He_*n*_^+^ at *n* = 74 or *n* = 58 when quantum effects are included. Independent PIMD simulations^53^ of C_60_He_*n*_^+^ found that the addition of further helium atoms beyond *n* = 32 induces significant disorder in the 1 × 1 commensurate phase, forming a liquid-like layer, which becomes rigid-like once again at *n* = 60 and is finally complete at *n* = 72. Harnisch et al. performed a study of helium-decorated fullerene anions^54^ and found that magic numbers observed for C_60_He_*n*_^–^ and C_70_He_*n*_^–^ are identical with their respective cationic counterparts, except for the second anomaly of C_70_He_*n*_^–^, which is found at *n* = 65 rather than *n* = 62 as for C_70_He_*n*_^+^. So far, the reason for these differences between cationic and anionic systems remains unclear.

In a subsequent study, Kuhn et al. investigated photodissociation of C_60_He_*n*_^+^ upon electronic excitation for complexes containing up to *n* ∼ 100 He atoms.^19^ Line shifts were determined to be a function of the number of He atoms attached. A remarkably linear red-shift of ∼0.07 nm per He atom was observed up to *n* = 32, followed by a nonlinear blue-shift up to *n* = 60 and a less pronounced red-shift up to *n* = 80. A precise understanding of the structure and binding energies of helium-ion complexes is very valuable for the interpretation of action spectra since it can be used to extrapolate the observed shift as a function of the number of He atoms down to zero, i.e., the position of the gas-phase transition.^20^ A recent investigation by Gatchell et al. highlighted the interplay of mass spectrometry, action spectroscopy, and path-integral molecular dynamics (PIMD) simulations to address helium solvation of corannulene cations.^55^ A comprehensive theoretical study of helium coating of the three planar, cationic PAHs (pyrene, coronene, and circumcoronene) as well as benzene was performed by one of us using a similar computational methodology as in the cationic fullerene case.^56^ It was found in this study that the graphitic surfaces are covered first by relatively strongly bound helium atoms which form a solid-like layer where helium atoms are highly localized above the aromatic rings. Additional helium atoms then cover the peripheral regions in the molecular plane, displaying an intermediate, “slushy” character, before a liquid-like outer layer is formed around the molecule. Shortly after, Kurzthaler et al. published an experimental study of helium coverage of coronene cations,^57^ identifying prominent stepwise intensity drops in the abundance of C_24_H_12_He_*n*_^+^ at *n* = 38, 41, and 44. These experimental results were in good agreement with a subsequent theoretical study of helium adsorption on neutral coronene performed by Rodríguez-Cantano et al., which reported enhanced stability of C_24_H_12_He_*n*_ at *n* = 38 (for both classical and quantum calculations) and completion of the first solvation shell at *n* = 44 (only evident in quantum calculations).^49^ Kurzthaler et al. thus proposed that in the *n* = 38 complex, all aromatic rings as well as the peripheral, open hexagons of coronene are covered by one helium atom each on both sides of the molecular plane, with three and six additional helium atoms added around the edge of the cation forming symmetrical structures of enhanced stability, explaining the stepwise drops at *n* = 41 and 44. These results were further confirmed by an additional PIMD study of the cationic system,^58^ in which it was also shown that the additional atoms beyond *n* = 38 appeared to be strongly delocalized rather than forming well-defined, rigid-like structures. Moreover, this study suggested that *n* = 44 does not correspond to the filling of the first solvation shell, as 50 helium atoms could still be inserted in a single solvation shell, thereby possibly revealing interesting differences with the neutral case^49^ and indicating that the anomalies in the experiment may not necessarily be associated with the completion of geometric shells for these hydrocarbon cations. In the present work, we further explore the solvation of cationic PAHs in helium by studying smaller PAHs than previously investigated and, for the first time, including isomeric species. We chose four PAHs with planar geometry: anthracene (Ant) and phenanthrene (Phe), both C_14_H_10_, as well as fluoranthene (Flu) and pyrene (Pyr), both C_16_H_10_. We demonstrate the sensibility of helium solvation characteristics to differences in the structure of the solvated PAH cation and further aim to emphasize the importance of interplay between experimental and theoretical work required to resolve the structure of helium-ion complexes.

## 2 Methods

### 2.1 Experimental Section

The experiment used for this study recently underwent reconstruction, significantly changing the ion production and ion extraction mechanisms to the point that the two setups can be considered different experiments. Detailed descriptions of the original^59^ (used for fluoranthene and pyrene) and the modified setup^10^ (used for anthracene and phenanthrene) can be found elsewhere; hence, we only give a brief outline here. In both experiments, a beam of HNDs with a mean size of typically a few million He atoms^60,61^ is produced by expanding precooled, pressurized (20–31 bar) 99.9999% purity helium through a nozzle of 5 μm in diameter cooled to 8.3–9.5 K. In the original setup, sample molecules are evaporated from a heated oven and picked up by (or doped into) neutral HNDs. The doped, neutral droplets are subjected to electron ionization (EI), resulting in the ejection of small ions and small ionic complexes from the droplets. In the modified setup, the HND beam is subjected to EI first, leading to the formation of highly charged droplets with an estimated average charge state of +10*e*.^61^ The highly charged droplets subsequently pick up sample molecules that are introduced into the pick-up region from an external heated reservoir via a heated stainless steel tube. The dopant molecules locate at the charge centers of the droplets and are ionized upon charge transfer from .^62^ The beam of highly charged, doped droplets then collides with a polished stainless steel surface, resulting in the ejection of ions. This method produces a much greater yield of ions complexed with up to ∼100 He atoms in comparison to the original setup.^63^ In both experiments, ions are guided from the ion extraction region into a high-resolution (*m*/Δ*m* = 1500–4500) reflectron time-of-flight mass spectrometer (ToF-MS) via weak electrostatic fields. Recorded mass spectra were analyzed by using the IsotopeFit software.^64^ All PAH samples were purchased from Sigma-Aldrich and have purities between 98 and 99.5%.

### 2.2 Computational Section

The same methodology already employed in an earlier investigation^56^ is followed here. Briefly, the cationic PAHs are treated as rigid, with their geometries optimized by using a standard quantum chemistry calculation at the level of density-functional theory using the B3LYP hybrid functional and the 6-31G(d,p) basis set.^65^ The atomic charges needed to represent the electrostatic environment felt by the helium atoms were obtained by the conventional RESP procedure.^66^ All optimized geometries with the corresponding set of partial charges are provided as Supporting Information. The classical and quantum structures of helium clusters around these cations were simulated at the atomistic level of details, assuming the very same polarizable potential already described in ref (56). Basin-hopping global optimization^67^ was first conducted for all clusters containing up to 50 helium atoms by using five series of 100000 random collective moves and an effective temperature of 10 K for the Metropolis Monte Carlo acceptance rates. Nuclear delocalization was then included through the PIMD methodology by performing trajectories of 1 ns, of which only the last 500 ps were considered for accumulating the properties, with a time step of 0.5 fs and a Trotter delocalization number of 256, the temperature of 1 K being maintained by means of massive Nosé–Hoover thermostats. The PIMD simulations were initiated from the putative classical global minima, and periodic quenches from the centroid positions were also performed to confirm that no new important minimum was missed. In the following, we denote by *E*_C_(*n*) the binding energy of the putative global minimum associated with the structure of *n* helium atoms around a given cationic PAH and refer to it as the classical energy. From those simulations the quantum energies *E*_Q_(*n*) were extracted from the virial estimator, and helium density plots were accumulated as well. The radial density measuring the geometrical distance between helium atoms and the nearest atom from the PAH cation was also determined but was not found to be very sensitive toward the completion of the first solvation shell because of excessively broad distributions.

From the classical (C) and quantum (Q) energies, first and second energy differences were also determined to provide a connection with experimentally measured ion abundances. Here we will particularly focus on the former quantity evaluated as
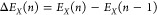
1where *X* = C or Q. To describe structures more conveniently, we will also refer to them using the notation *X* + *Y* (+ *Z*), where *X* and *Y* denote the number of helium atoms lying on either side of the PAH and *Z*, if present, denotes the number of atoms lying in the vicinity of the PAH plane. In addition to the classical and quantum structures and their associated energies, we also explored the extent of statistical delocalization in the nuclear wave function probed by the PIMD simulations, by performing regular quenches from the instantaneous centroid positions. Doing so provides a number of locally stable isomers {*i*} with classical energies {*E*_*i*_}, each of them being found a number of times, giving, after normalization by the number of quenches performed, an occurrence probability *p*_*i*_. From the set of probabilities {*p_i_*}, an information entropy *S*_IS_ associated with these inherent structures can be evaluated as *S*_IS_ = −*k*_B_*∑*_*i*_*p*_*i*_ ln(*p*_*i*_), where *k*_B_ is the Boltzmann constant. *S*_IS_/*k*_B_ is a dimensionless number that measures the structural diversity hidden in the quantum wave function and is vanishing if and only if the wave function is associated with a single well-defined structure, which may or may not be the classical minimum.^68^ Here we use this quantity as a delocalization index measuring the extent to which the classical and quantum structures differ from one another, from a statistical perspective.

## 3 Results and Discussion

### 3.1 Mass Spectra

**Figure 1:**
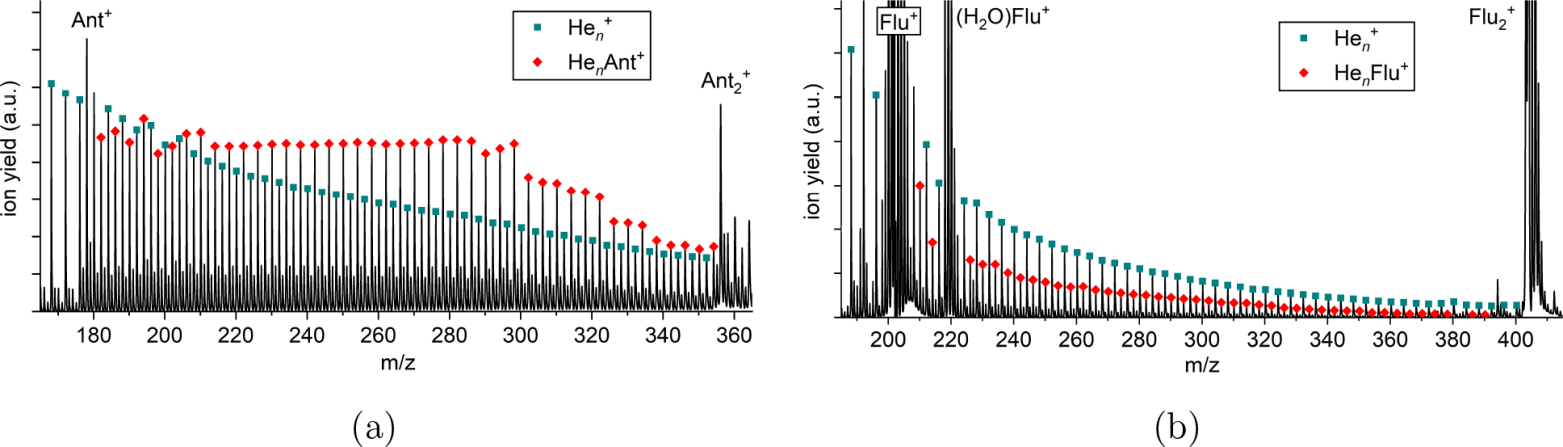
Time-of-flight mass spectra of cationic helium–PAH complexes obtained by doping HNDs with the PAHs (a) anthracene, recorded with the modified setup, and (b) fluoranthene, recorded with the original setup. Both mass spectra reveal ion series of bare helium clusters  (turquoise squares) and helium-decorated cations He_*n*_PAH^+^ (red diamonds), alongside contributions of bare and water-decorated PAH clusters. Differences in the overall shape of the He_*n*_PAH^+^ distribution and the relatively higher yield of He_*n*_Ant^+^ are caused by the different ionization and ion extraction mechanisms (see the Experimental Section).

Figure 1 shows excerpts of two time-of-flight mass spectra of HNDs doped with fluoranthene (panel b, recorded with the original setup) and anthracene (panel a, recorded with the modified setup). Both mass spectra extend from the respective monomer to the dimer region. The strongest signals in the fluoranthene spectrum are caused by the bare monomer and dimer as well as the monomer complexed with one water molecule picked up from the residual gas and are between 1 and 3 orders of magnitude stronger than the ion yields from He-tagged fluoranthene ions. Unfortunately, some of the peaks corresponding to He-tagged fluoranthene ions are obscured by isobaric ions, caused by isotopes of the bare monomer or oxygenated fluoranthene, the latter most likely being a fragment of the abundant fluoranthene–water complex. In the region between the monomer and dimer, two weaker ion series can be found that are spaced by ∼4 *m*/*z* and exhibit an approximately exponential decrease with increasing *m*/*z*. These two series can be attributed to bare helium clusters  and fluoranthene monomer cations complexed with up to ∼50 helium atoms, He_*n*_Flu^+^. Equivalent signals can be found in the anthracene mass spectrum, but with very different relative intensities, which can be attributed to the different mechanisms of dopant ionization and extraction of ions from the helium droplets.^10,63^ The bare monomer and dimer signals are still among the strongest ion signals but much weaker compared to the fluoranthene mass spectrum. The (H_2_O)Ant^+^ signal is so weak that it cannot be distinguished in the presented figure. In contrast, the He_*n*_Ant^+^ series is notably stronger and no longer exhibits an apparent exponential decrease with increasing *n*, but rather similar ion yields between *n* = 1–30, followed by a decline in intensity above *n* = 30. Several anomalies like pronounced local maxima and stepwise drops of the ion yield can be clearly noticed at specific numbers *n*. Similar features are also found in the He_*n*_Flu^+^ series but are less apparent due to the lower ion yield. Intensity anomalies in the progression of He_*n*_PAH^+^ originate from changes in the respective dissociation energies of those complexes. Complexes with lower dissociation energies are more easily dissociated, and thus the corresponding ion yields are depleted, whereas the ion yields of particularly stable complexes appear relatively enhanced, leading to the observed local anomalies. We distinguish between two types of local anomalies detected in the experimental data: magic numbers, characterized by an anomalously high ion yield compared to both *n* – 1 and *n* + 1, as well as stepwise drops, characterized by a significantly higher ion yield compared to *n* + 1 but not *n* – 1. It should be noted that these designations can be subjective and ambiguous in some cases, which is why we focus only on the more prominent features. While the He_*n*_PAH^+^ series extend to *n* ∼ 100 for all studied PAHs, the region *n* < 40 is most interesting for our analysis since it contains almost all of the detected features. In the following sections, we will analyze the information extracted from mass spectra in combination with computational results and thus contribute to determining the structure of the cationic PAHs anthracene, phenanthrene, fluoranthene, and pyrene decorated with finite numbers of helium atoms.

### 3.2 Anthracene

**Figure 2:**
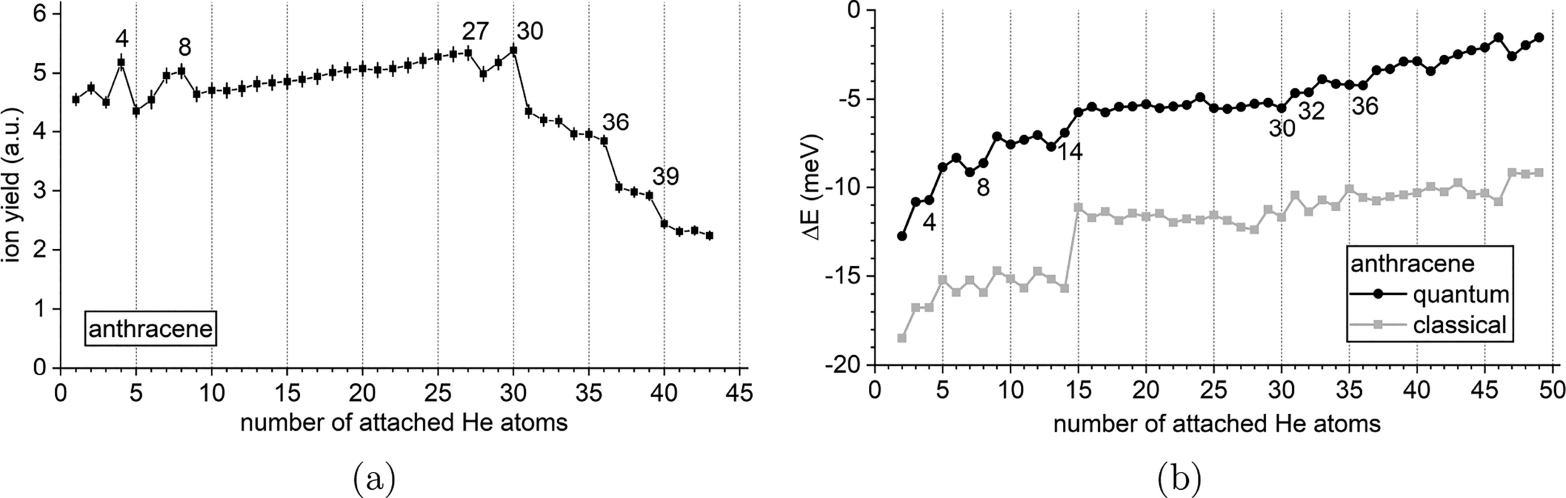
(a) Experimental distribution of He_*n*_Ant^+^ as a function of *n*. Magic numbers are found at *n* = 4 and 8 with additional stepwise drops at *n* = 27, 30, 36, and 39. (b) First energy differences Δ*E*(*n*) calculated from the classical global minima (gray symbols) or from the quantum virial energies (black symbols).

**Figure 3:**
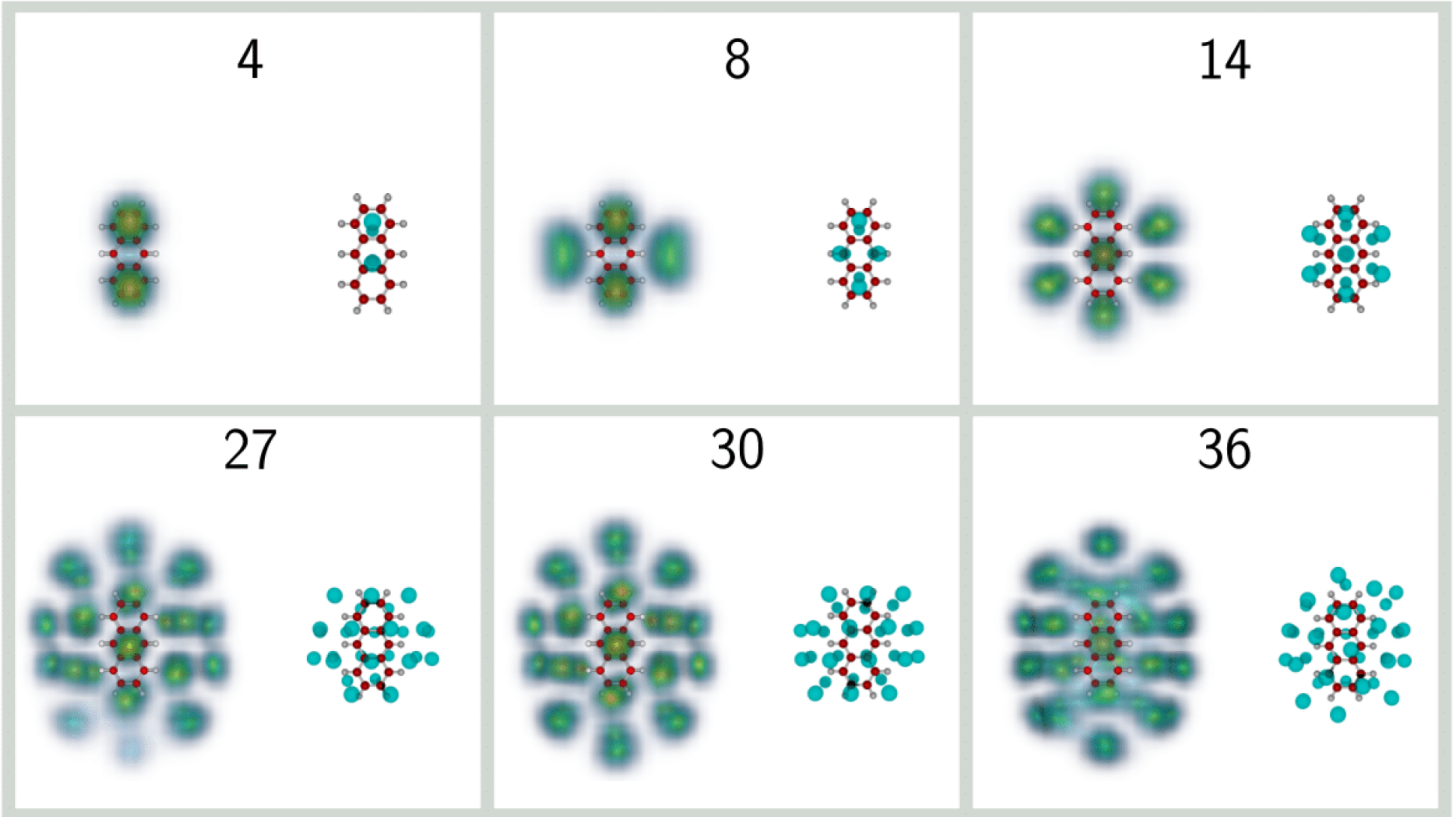
Selected structures of He_*n*_Ant^+^ clusters with *n* = 4, 8, 14, 27, 30, and 36. For each size, the classical global minima are shown on the right, while the helium densities obtained from the PIMD simulations are shown on the left.

Figure 2a shows the ion yield of the He_*n*_Ant^+^ series (cf. Figure 1a) as a function of *n* from 1 to 43 attached helium atoms. As already pointed out, the ion yield remains similar up to *n* = 30, followed by a steep decline. We immediately identify prominent magic numbers at *n* = 4 and 8 as well as stepwise drops at *n* = 27, 30, 36, and 39. In Figure 2b, the first energy differences Δ*E*(*n*) obtained in both classical and quantum descriptions are shown. Whereas the steps in Δ*E*(*n*) at *n* = 4, 8, 30, and 36 coincide with experimentally detected anomalies, no pronounced features corresponding to the experimentally detected anomalies at *n* = 27 and 39 are found. The calculations further suggest the existence of anomalies at *n* = 14 and 32, which are not reflected in the mass spectra. Figure 3 depicts the corresponding classical and quantum structures obtained for selected sizes, from the putative global minima or the nuclear densities sampled from the PIMD trajectories, respectively. The growth pattern proceeds similarly as previously discussed for coronene^49,56,58^ and consists first of the adsorption of helium atoms on both sides of the carbon rings (2 + 2 pattern), then of the filling of the peripheral region closer to the PAH plane, until the shell is complete. Shell filling itself is not a sharp process but occurs in a range that depends on the number of atoms needed to fill this peripheral region and in which the binding energy varies more smoothly.

In general, the nuclear density is significantly more extended than the space covered by the classical positions, as a result of vibrational delocalization. Such an effect often produces nuclear wave functions that do not present the exact same symmetric features as those exhibited by the classical structure. An archetypal example is provided for the *n* = 4 complex, for which the classical minimum has two helium atoms adsorbed on both sides of the PAH, near adjacent aromatic rings, in a commensurate 1 × 1 fashion. Once nuclear delocalization is accounted for, the two atoms on either side expand and preferentially occupy the outer rings. Such an expansion is also manifested for the *n* = 8 complex, but now in a more lateral fashion for this cluster that adopts a 4 + 4 adsorption pattern, keeping the symmetry in the quantum case. At size 14, a double hexagonal filling 7 + 7 is predicted, also closer to the  commensurate filling of helium monolayers adsorbed on graphite, in which half of the hexagonal sites are occupied.

**Figure 4:**
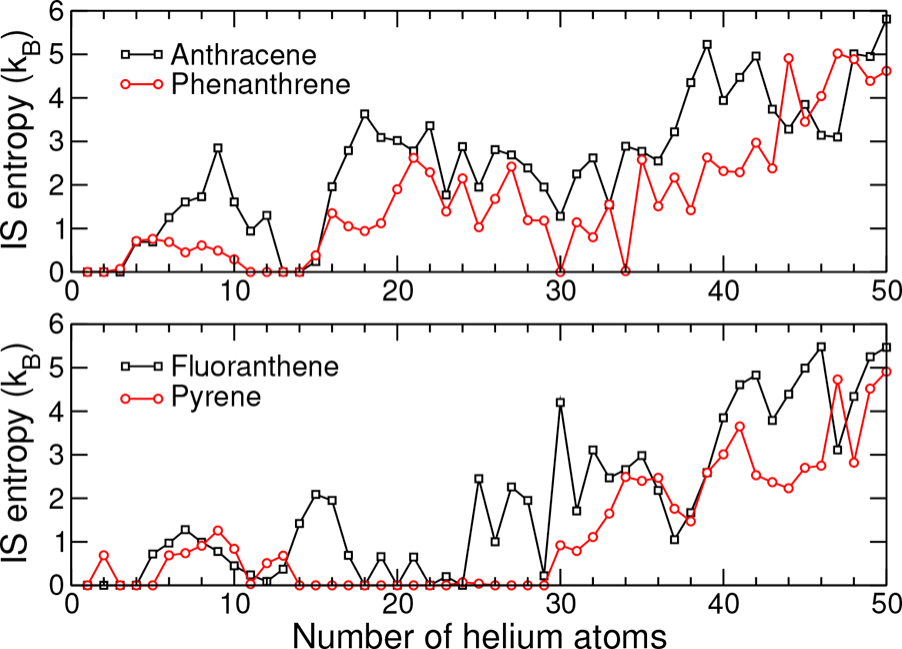
Inherent structure entropy *S*_IS_ obtained from the PIMD trajectories of the He_*n*_ clusters coating the anthracene, phenanthrene, fluoranthene, and pyrene cations as a function of *n*.

This size is predicted to be particularly stable in the classical case, with not only a strong step in Δ*E* but also a drop in the inherent structure entropy *S*_IS_, whose variations with increasing size are depicted in Figure 4.

Interestingly, the delocalization index *S*_IS_ is strictly positive for most other sizes, including those that are found to lead to prominent steps in the first energy difference. As will be seen below, a similar behavior is found to be relatively general and hold for most of the studied PAH cations.

As more helium atoms are added, the solvation shell further expands, but the central hexagonal motif remains and imposes further growth. The structure obtained at size 27 is not particularly symmetric and reveals an incomplete outer shell. Our calculations indicate that shell filling begins at size 30 and ends below size 36, the cluster at this size already exhibiting some significant degree of fluxionality, with the nuclear density no longer showing well-defined spots. Noteworthily, at these sizes of 30 and 36 the quantum structures display a higher symmetry degree than their classical counterparts, which also indirectly confirms their greater stability suggested by the mass spectra. We thus interpret shell filling around cationic anthracene to occur in the approximate range of 30–36 attached helium atoms.

### 3.3 Phenanthrene

**Figure 5:**
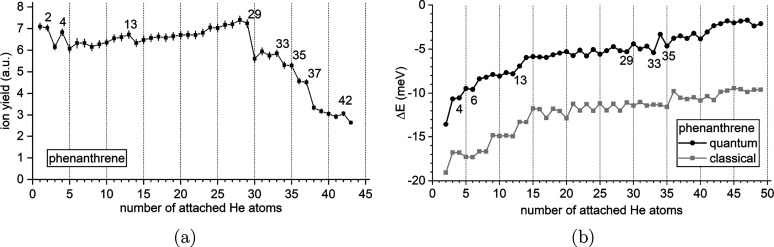
(a) Experimental distribution of He_*n*_Phe^+^ as a function of *n*. Magic numbers are seen at *n* = 4 and 42, with additional stepwise drops at *n* = 2, 13, 29, 33, 35, and 37. (b) First energy differences Δ*E*(*n*) calculated from the classical global minima (gray symbols) or from the quantum virial energies (black symbols).

Figure 5a shows the He_*n*_Phe^+^ series as a function of *n* from 1 to 43 attached He atoms. The overall shape is very similar to that of He_*n*_Ant^+^. Prominent magic numbers are found at *n* = 4 and a weaker one at *n* = 42 as well as stepwise drops at *n* = 2, 13, 29, 33, 35, and 37. Whereas the overall shape of the distribution is very similar to that of He_*n*_Ant^+^, the anomalies exhibit a different pattern. Both compounds share the first magic number at *n* = 4; however, there is no equivalent to the local maximum of He_*n*_Ant^+^ at *n* = 8 in the He_*n*_Phe^+^ series. Instead, a pronounced step is found at *n* = 13, which itself has no equivalent in the He_*n*_Ant^+^ series. The other features are qualitatively similar, with the most prominent step occurring at *n* = 29 for He_*n*_Phe^+^ (instead of *n* = 30 for He_*n*_Ant^+^), followed by a series of smaller steps. The smaller stepwise drops are spaced by two helium atoms for He_*n*_Phe^+^ instead of three for He_*n*_Ant^+^. A feature similar to the step at *n* = 27 in He_*n*_Ant^+^ is missing in the He_*n*_Phe^+^ series. In Figure 5b, the calculated first energy differences Δ*E*(*n*) for He_*n*_Phe^+^ are shown. The overall agreement between the progression of He_*n*_Phe^+^ and Δ*E*(*n*) is good, with important features such as the anomalies at *n* = 2, 4, 13, 29, 33, and 35 being correctly captured by steps in the quantum calculations. The prominent step at *n* = 37 is not reproduced well by theory but instead expected at *n* = 38. Also, some intermediate features are predicted at *n* = 6, 21, and 23 but are not particularly reflected in the mass spectra.

**Figure 6:**
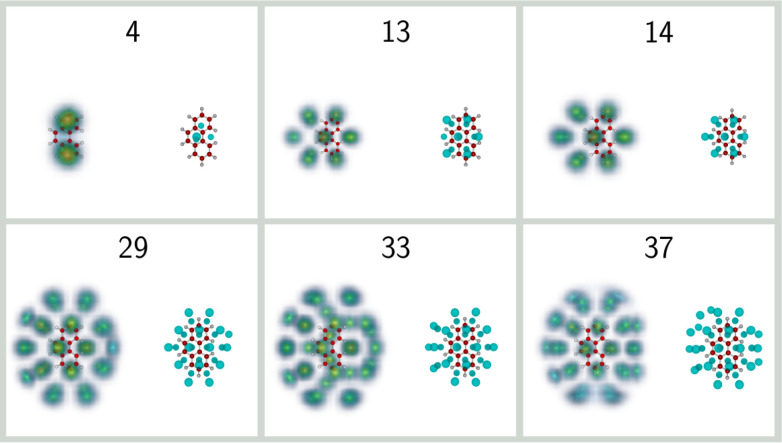
Selected structures of He_*n*_Phe^+^ clusters with *n* = 4, 13, 14, 29, 33, and 37. For each size, the classical global minima are shown on the right, while the helium densities obtained from the PIMD simulations are shown on the left.

The corresponding classical and quantum structures obtained for selected sizes of He_*n*_Phe^+^ are shown in Figure 6. The different positions of the outer hexagons relative to anthracene give rise to interesting differences in physical adsorption patterns. For this PAH, the classical and quantum structures obtained for the *n* = 4 complex are at qualitative variance, being of the 3 + 1 and 2 + 2 types, respectively, as the result of competing zero-point energies. In the quantum case, the atoms are preferentially adsorbed on either side of the outer hexagons. Upon adding helium atoms, the same growth mechanisms as in the anthracene case seem to be followed, ending up at size 14 as the 7 + 7 hexagonal motif. However, the different topology of phenanthrene makes it energetically preferable to adsorb 13 atoms only, as 6 + 6 and one atom near the PAH plane (in average) and on its concave side. The extra atom leading to the more localized 7 + 7 system is thus found to be also more strained and less favorable. The lower symmetry of phenanthrene is also reflected on the structure of larger clusters, particularly at size 33 where all helium atoms still manage to occupy rather localized sites. From Figure 4, inherent structures also appear to be very few at size 34 for this cation, indicating very similar classical and quantum structures and a much reduced degree of delocalization. In contrast, at size 37 the extent of vibrational delocalization has become significant and the solvation shell appears disrupted. For the phenanthrene cation, we evaluate the shell filling range as taking place between 29 and 34 He atoms.

### 3.4 Fluoranthene

**Figure 7:**
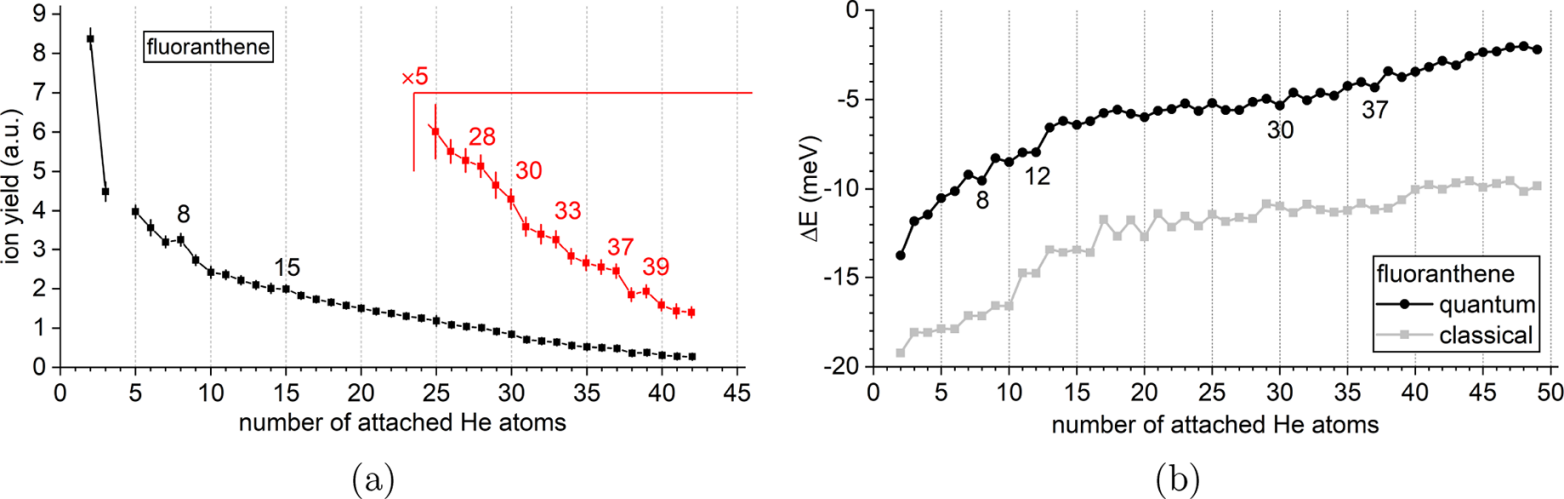
(a) Experimental distribution of He_*n*_Flu^+^ as a function of *n*. Magic numbers are seen at *n* = 8, 15, 28, and 39, with additional stepwise drops at *n* = 30, 33, and 37. Missing data points are caused by the interference of isobaric ions (see discussion in Figure 1b). (b) First energy differences Δ*E*(*n*) calculated from the classical global minima (gray symbols) or from the quantum virial energies (black symbols).

Figure 7a displays the distribution of He_*n*_Flu^+^ from *n* = 2 to 42. The He_*n*_Flu^+^ series exhibits a lower overall intensity and a different shape compared to both the He_*n*_Ant^+^ and He_*n*_Phe^+^ series due to the different experimental setups (see the Experimental and Mass Spectra sections). The ion yield is highest for the *n* = 2 complex and declines rapidly with increasing *n*, loosely resembling an exponential decay. Magic numbers are not immediately obvious but can be found upon closer inspection at *n* = 8, 15, 28, and 39, with stepwise drops occurring at *n* = 30, 33, and 37. A comparison with the calculated Δ*E*(*n*) displayed in Figure 7b shows reasonable agreement, with the prominent features at *n* = 8, 30, and 37 being well-captured by the calculations. However, other features do not coincide with local maxima in Δ*E*(*n*), the calculations notably predicting a particularly stable structure at *n* = 12 that is not reflected in the experimental data.

**Figure 8:**
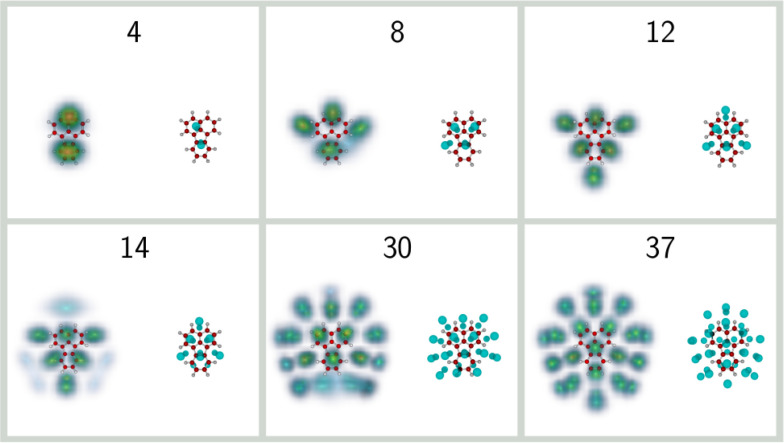
Selected structures of He_*n*_Flu^+^ clusters with *n* = 4, 8, 12, 14, 30, and 37. For each size, the classical global minima are shown on the right, while the helium densities obtained from the PIMD simulations are shown on the left.

The corresponding classical and quantum structures obtained for selected sizes are shown in Figure 8 for this PAH. The peculiar topology of fluoranthene gives rise to also rather specific adsorption preferences. At both sizes 4 and 8, the helium atoms are evenly shared on both sides of the PAH and either adsorbed on next-neighbor hexagonal rings (*n* = 4 case) or more closely packed toward the two adjacent hexagonal rings (*n* = 8 case), but always producing classical structures that are lower in symmetry than the PAH itself. Once quantum effects are accounted for the significant expansion of the nuclear wave function restores this symmetry, preferred adsorption sites now being the single hexagonal ring and opposite sites lying away from the molecule (see Figure 8). The more difficult accommodation of the adsorbed helium atoms on the fluoranthene topology is particularly obvious at size 12, which despite still being of the 6 + 6 type shows a rather spectacular inversion between the classical and quantum structures. The structure obtained at *n* = 14 builds on the same pattern as the *n* = 12 complex, but differently in the classical and quantum cases, where its adsorption pattern should be better described as 6 + 8 or 6 + 6 + 2, respectively, two extra floating atoms exhibiting strong delocalization in the quantum structure. At size 30 a markedly different adsorption pattern is found, in which a 5-fold symmetry develops, still with an incomplete shell, ending at size 37 as the 16 + 16 + 5 quantum structure. Here each 16-atom arrangement can also be described as one central atom surrounded by two pentagonal rings. This 5-fold symmetric structure, which is also at variance with the classical minimum, results from a non trivial combination of the natural hexagonal packing preferred by helium monolayers and the specific topology of the fluoranthene cation. It is also highly localized and is associated with a low entropy of its inherent structures (see Figure 4). With regard to now shell completion around the fluoranthene cation, we roughly evaluate it to occur in the range of 31–37 attached helium atoms based on the results obtained around these sizes.

### 3.5 Pyrene

**Figure 9:**
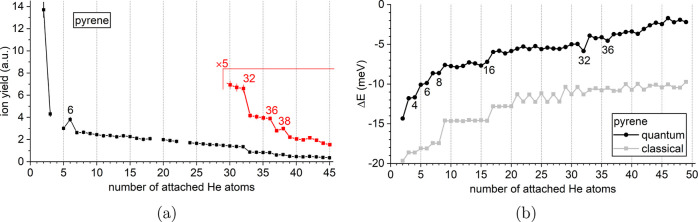
(a) Experimental distribution of He_*n*_Pyr^+^ as a function of *n*. Magic numbers are seen at *n* = 6 and 38, with additional stepwise drops at *n* = 32 and 36. Missing data points are caused by the interference of isobaric ions (see discussion of Figure 1b). (b) First energy differences Δ*E*(*n*) calculated from the classical global minima (gray symbols) or from the quantum virial energies (black symbols).

The He_*n*_Pyr^+^ series shown in Figure 9a exhibits a similar overall shape as the He_*n*_Flu^+^ series, but with a steeper decline from *n* = 2 to 3 and a slower, more linear decrease for *n* ≥ 7. Local anomalies are easily identified with a pronounced magic number at *n* = 6 and a weaker one at *n* = 38 as well as two stepwise drops: a prominent one at *n* = 32 and a weaker one at *n* = 36. The strongest feature in the calculated Δ*E*(*n*), shown in Figure 9b, is the strong local minimum at *n* = 32 which coincides with the most prominent step observed in the mass spectrum. The magic number at *n* = 6 is also captured by the calculations. However, the calculations do not account for the weaker step observed at *n* = 36 and the magic number at *n* = 38 but instead predict a particularly stable complex at *n* = 16 which is not reflected in the experimental data.

**Figure 10:**
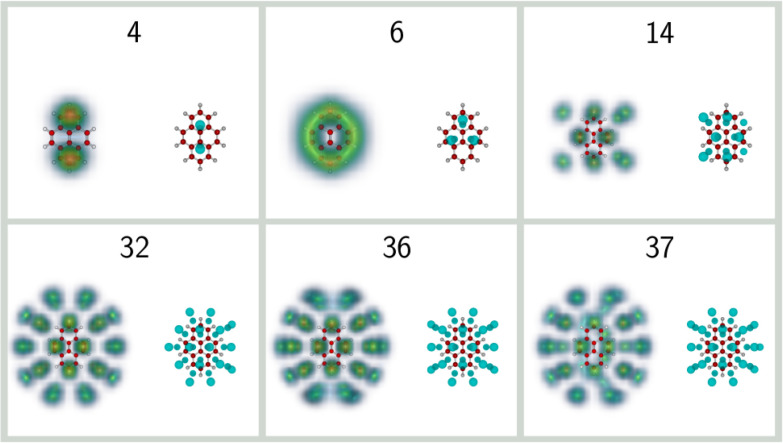
Selected structures of He_*n*_Pyr^+^ clusters with *n* = 4, 6, 14, 32, 36, and 37. For each size, the classical global minima are shown on the right, while the helium densities obtained from the PIMD simulations are shown on the left.

The corresponding classical and quantum structures obtained for selected sizes of He_*n*_Pyr^+^ are shown in Figure 10. The *n* = 4 complex adopts a 2 + 2 structure in both classical and quantum cases, with adsorption above the two outermost hexagonal rings. Additional helium atoms tend to occupy lateral positions, forming first a particularly stable 4 + 4 structure which further evolves into the 8 + 8 structure commensurate with the  pattern at size 16. At the intermediate size 14, this pattern is incomplete and is found as 6 + 8 in both classical and quantum cases. A more interesting case is that of He_6_Pyr^+^, which is found as a magic number in both experiment and calculations. While this system classically adopts a 3 + 3 structure, quantum effects produce a particularly delocalized wave function where the helium atoms occupy two oval rings on either side of the planar cation. Such an enhanced delocalization originates from the easy lateral motion of helium on the corrugated PAH surface and was also noted in related systems such as He_10_coronene^+^ ^56^ for the exact same reason. As seen from Figure 4, it is associated with multiple inherent structures differing in which three aromatic rings are being occupied on each side. One remarkable result for the He_6_Pyr^+^ complex is that nuclear delocalization is again responsible for the quantum structure exhibiting a higher symmetry than the classical counterpart, consistent with the increased stability suggested by the experimental data. Noteworthily, none of the other *n* = 6 clusters obtained for the anthracene, phenanthrene, or fluoranthene cations display such highly delocalized helium densities, despite the classical structures being also generally of the 3 + 3 type (2 + 4 for anthracene).

For the pyrene cation, complexes with between 14 and 29 attached helium atoms are all found to be associated with a zero inherent structure entropy, indicating that the corresponding nuclear wave functions in this size range are all associated with a well-defined local minimum, hence a single geometric structure. This behavior is specific to this molecule, and indicates a more regular type of atomic arrangement produced by pyrene, in contrast with the more disordered structures obtained around the other three cations when 14 ≤ *n* ≤ 29. Such differences are also consistent with the marked differences observed in the experimental ion abundances.

In larger complexes, the  core motif is preserved upon further addition of helium atoms toward the completion of the first shell. A particularly symmetric quantum structure is thus obtained at *n* = 32 as 14 + 14 + 4, and another one at *n* = 36 as 14 + 14 + 8, only the latter also showing a symmetric classical structure. Adding yet another helium atom at *n* = 37 disrupts this symmetry, and we conclude that based on our calculations, shell filling takes place in the range of 32—36 attached helium atoms for the pyrene cation.

## 4 Summary and Conclusion

The employed experimental and computational methods revealed a unique set of local anomalies for each of the investigated PAHs in the respective He_*n*_PAH^+^ ion series, summarized in Table 1. The best agreement between experiment and theory is found when nuclear delocalization is accounted for by using PIMD simulations, which are capable of revealing features that are not recognized by classical methods.

**Table 1:**
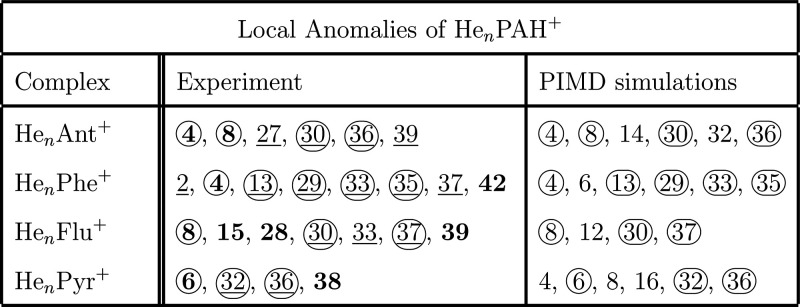
Local Anomalies of He_*n*_PAH^+^ Complexes Found via Experiment and PIMD Simulations Employed in This Study^a^

a Experimentally found anomalies are categorized as magic numbers (bold) and stepwise drops (underlined). Circled numbers represent anomalies found simultaneously in the experiment and PIMD simulations.

While the arrangement of adsorbed helium atoms is found to be very sensitive toward the structure of the solvated PAH cation, there are also common adsorption patterns between the studied molecules. For these rather small and planar polyaromatic solutes, helium atoms preferentially adsorb following the  commensurate pattern, rather than the 1 × 1 fashion found for larger species such as coronene^57,58^ as well as the curved corannulene^55^ or fullerenes.^51,53^ In the latter two cases, the curvature of the molecules facilitates the 1 × 1 adsorption. For the planar coronene, however, this behavior can be attributed to the specific size of this molecule, as we expect much larger PAHs to adsorb similarly as graphitic structures, i.e., as . We anticipate a similar evolution triggered by decreasing curvature in the giant fullerenes, although those are known not to remain spherical after a while. It would be interesting to investigate PAHs larger than coronene to see if and at what size a crossover to the  pattern can be found or whether different factors are responsible for the 1 × 1 adsorption pattern observed for coronene.

Our PIMD simulations also reveal that zero-point effects are qualitatively important in explaining many of the microsolvated structures. In particular, several quantum structures are found to exhibit a higher symmetry than the corresponding classical global minima, made possible by the expansion of the nuclear wave function. This occurs e.g. for He_32_Ant^+^, He_4_Phe^+^, He_13_Phe^+^, He_8_Flu^+^, He_37_Flu^+^, or He_32_Pyr^+^ and was also noted earlier in the case of not only He_38_Coronene^+^ ^58^ but also chemically different systems such as .^69^

A manifestation of the importance of quantum nuclear effects is found, for most clusters, in the diversity of inherent structures that are probed by the centroids in the PIMD trajectories, as shown by strictly positive information entropies. Even particularly stable clusters in the first energy difference are associated with multiple local minima in the energy landscape, which typically differ from each other by the specific location of the helium atoms near the peripheral region. Contrary to cationic neon clusters,^70^ we found no evidence here for higher stabilities in the abundances to be associated with a particularly low vibrational delocalization index, as measured by the inherent structure entropy. However, the marked sensitivity to the specific PAH structure found in our measurements is well-reflected in not only the strong differences between the solvation patterns exhibited by the nuclear densities but also the inherent structure entropies obtained between the anthracene and phenanthrene cationic solutes and those obtained between the fluoranthene and pyrene cationic solutes.

Residual discrepancies between the experimental data and our simulations could point at some approximations in our modeling that would need to be overcome in future computational developments. Besides the neglect of bosonic exchange, the rigid treatment of the hydrocarbon could be excessively simplistic, especially in the peripheral region of the PAHs where the terminating hydrogen atoms are likely prone to quantum delocalization themselves. While exchange statistics might not be essential for the present hydrocarbon solutes,^71^ to which helium binds rather strongly, a flexible model would probably be more realistic in describing clusters near shell closure, as suggested by the recent work from the Marx group on the (strongly fluxional and rather extreme) case of protonated methane.^72^ To assess the importance of hydrogen delocalization and evaluate the validity of the rigid approximation used here for the cationic solutes, experiments on deuterated species could be particularly useful. We also plan to address the influence of helium solvation patterns toward spectra of electronic and vibrational transitions as observed via action spectroscopy of helium-solvated compounds in a future publication.

## Supporting Information Available

Detailed geometries and atomic charges for the cationic PAHs (PDF)
