# Supplementary material for: Adsorption of Helium on Small Cationic PAHs: Influence of Hydrocarbon Structure on the Microsolvation Pattern
Source: J Phys Chem A. 2021 Aug 26;125(36):7813–24. doi: 10.1021/acs.jpca.1c05150 (PMC8450901; doi:10.1021/acs.jpca.1c05150)
Supplement: Supplementary file 1 — jp1c05150_si_001.pdf [file jp1c05150_si_001.pdf]

# Electronic Supplementary Information for "Adsorption of Helium on Small Cationic PAHs: Influence of Hydrocarbon Structure on the Microsolvation Pattern"

Arne Schiller,[*,†] Miriam Meyer,[†] Paul Martini,[†] Fabio Zappa,[†] Serge A. Krasnokutski,[‡] Florent Calvo,[¶] and Paul Scheier[†]

†*Institut für Ionenphysik und Angewandte Physik, Universität Innsbruck, Technikerstr. 25, A-6020 Innsbruck, Austria*

‡*Laboratory Astrophysics roup of the MPI for Astronomy at the University of Jena, Hemlholtzweg 3, D-07743 Jena, Germany*

¶*Université Grenoble Alpes, CNRS, LiPhy, F-38000 Grenoble, France*

E-mail: arne.schiller@uibk.ac.at

# Contents

1. Atomic structures of the cationic PAHs used in the modeling

# 1 Atomic structures of the cationic PAHs used in the modeling

The five columns indicate the atom types, $x$, $y$, and $z$ positions, and the atomic charge $q$, respectively.

## 1.1 Anthracene cation

$C_{14}H_{10}^{+}$:

| | | | | |
|---|---|---|---|---|
| C | 0.000000 | 1.405755 | 0.0000 | -0.222944 |
| C | 0.000000 | -1.405755 | 0.0000 | -0.222944 |
| C | 1.232322 | 0.720277 | 0.0000 | 0.144208 |
| C | 1.232322 | -0.720277 | 0.0000 | 0.144208 |
| C | -1.232322 | -0.720277 | 0.0000 | 0.144208 |
| C | -1.232322 | 0.720277 | 0.0000 | 0.144208 |
| C | 2.468598 | 1.407655 | 0.0000 | -0.122359 |
| C | 2.468598 | -1.407655 | 0.0000 | -0.122359 |
| C | -2.468598 | -1.407655 | 0.0000 | -0.122359 |
| C | -2.468598 | 1.407655 | 0.0000 | -0.122359 |
| C | 3.667624 | 0.702767 | 0.0000 | -0.059847 |
| C | 3.667624 | -0.702767 | 0.0000 | -0.059847 |
| C | -3.667624 | -0.702767 | 0.0000 | -0.059847 |
| C | -3.667624 | 0.702767 | 0.0000 | -0.059847 |
| H | 0.000000 | 2.492755 | 0.0000 | 0.182012 |

| | | | | |
|---|---|---|---|---|
| H | 0.000000 | -2.492755 | 0.0000 | 0.182012 |
| H | 2.473316 | 2.493442 | 0.0000 | 0.154540 |
| H | 2.473316 | -2.493442 | 0.0000 | 0.154540 |
| H | -2.473316 | -2.493442 | 0.0000 | 0.154540 |
| H | -2.473316 | 2.493442 | 0.0000 | 0.154540 |
| H | 4.609829 | 1.240519 | 0.0000 | 0.153924 |
| H | 4.609829 | -1.240519 | 0.0000 | 0.153924 |
| H | -4.609829 | -1.240519 | 0.0000 | 0.153924 |
| H | -4.609829 | 1.240519 | 0.0000 | 0.153924 |

## 1.2 Phenanthrene cation

$C_{14}H_{10}^{+}$:

| | | | | |
|---|---|---|---|---|
| C | 0.732691 | -0.397567 | 0.0000 | 0.102821 |
| C | -0.732691 | -0.397567 | 0.0000 | 0.102821 |
| H | 1.030515 | -2.538034 | 0.0000 | 0.132461 |
| C | -0.700377 | 2.061963 | 0.0000 | -0.109506 |
| C | 0.700377 | 2.061963 | 0.0000 | -0.109506 |
| H | -1.030515 | -2.538034 | 0.0000 | 0.132461 |
| C | 1.502526 | -1.562753 | 0.0000 | -0.199653 |
| C | -1.429505 | 0.860360 | 0.0000 | 0.117807 |
| C | 1.429505 | 0.860360 | 0.0000 | 0.117807 |
| C | -1.502526 | -1.562753 | 0.0000 | -0.199653 |
| C | 2.904449 | -1.508005 | 0.0000 | 0.055253 |
| C | -2.857609 | 0.889966 | 0.0000 | -0.088805 |
| C | 2.857609 | 0.889966 | 0.0000 | -0.088805 |
| C | -2.904449 | -1.508005 | 0.0000 | 0.055253 |
| C | 3.587271 | -0.281922 | 0.0000 | -0.109508 |

| | | | | |
|---|---|---|---|---|
| C | -3.587271 | -0.281922 | 0.0000 | -0.109508 |
| H | -1.233811 | 3.007937 | 0.0000 | 0.175618 |
| H | 1.233811 | 3.007937 | 0.0000 | 0.175618 |
| H | 3.466262 | -2.436702 | 0.0000 | 0.130131 |
| H | -3.357553 | 1.853811 | 0.0000 | 0.143806 |
| H | 3.357553 | 1.853811 | 0.0000 | 0.143806 |
| H | -3.466262 | -2.436702 | 0.0000 | 0.130131 |
| H | 4.671166 | -0.259266 | 0.0000 | 0.149575 |
| H | -4.671166 | -0.259266 | 0.0000 | 0.149575 |

## 1.3 Fluoranthene cation

$C_{16}H_{10}^{+}$:

| | | | | |
|---|---|---|---|---|
| C | -1.303557 | 0.681419 | 0.000000 | 0.079396 |
| C | -1.278976 | -0.750603 | 0.000000 | 0.118253 |
| C | -2.459136 | -1.461110 | 0.000000 | -0.122690 |
| C | -3.691736 | -0.746511 | 0.000000 | 0.014966 |
| C | -3.720543 | 0.641802 | 0.000000 | -0.146517 |
| C | -2.520228 | 1.374670 | 0.000000 | -0.047876 |
| C | 0.140324 | -1.179129 | 0.000000 | -0.120497 |
| C | 2.973580 | -1.214824 | 0.000000 | -0.107411 |
| C | 2.228441 | -2.395533 | 0.000000 | -0.137203 |
| C | 0.816369 | -2.399658 | 0.000000 | -0.034768 |
| C | 0.901341 | 0.000000 | 0.000000 | 0.207263 |
| C | 0.066501 | 1.156506 | 0.000000 | -0.082213 |
| C | 0.679683 | 2.425128 | 0.000000 | -0.024880 |
| C | 2.075061 | 2.487971 | 0.000000 | -0.132310 |
| C | 2.887187 | 1.330263 | 0.000000 | 0.007878 |

| | | | | |
|---|---|---|---|---|
| C | 2.303673 | 0.038837 | 0.000000 | 0.008526 |
| H | -2.468737 | -2.546544 | 0.000000 | 0.127209 |
| H | -4.622370 | -1.304453 | 0.000000 | 0.145473 |
| H | -4.670388 | 1.164608 | 0.000000 | 0.157624 |
| H | -2.544067 | 2.460102 | 0.000000 | 0.151145 |
| H | 4.058267 | -1.257259 | 0.000000 | 0.158117 |
| H | 2.751224 | -3.346125 | 0.000000 | 0.164587 |
| H | 0.285557 | -3.346461 | 0.000000 | 0.154841 |
| H | 0.095832 | 3.339758 | 0.000000 | 0.149747 |
| H | 2.559079 | 3.458945 | 0.000000 | 0.161141 |
| H | 3.967707 | 1.442060 | 0.000000 | 0.150199 |

## 1.4 Pyrene cation

$C_{16}H_{10}^{+}$:

| | | | | |
|---|---|---|---|---|
| C | 0.000000 | 0.708380 | 0.0000 | -0.015092 |
| C | 0.000000 | -0.708380 | 0.0000 | -0.015092 |
| C | 2.450763 | 0.692447 | 0.0000 | -0.131725 |
| C | -2.450763 | -0.692447 | 0.0000 | -0.131725 |
| C | -2.450763 | 0.692447 | 0.0000 | -0.131725 |
| C | 2.450763 | -0.692447 | 0.0000 | -0.131725 |
| C | 1.233843 | 1.420695 | 0.0000 | 0.140514 |
| C | -1.233843 | -1.420695 | 0.0000 | 0.140514 |
| C | -1.233843 | 1.420695 | 0.0000 | 0.140514 |
| C | 1.233843 | -1.420695 | 0.0000 | 0.140514 |
| C | 1.206579 | 2.843522 | 0.0000 | -0.105788 |
| C | -1.206579 | -2.843522 | 0.0000 | -0.105788 |
| C | -1.206579 | 2.843522 | 0.0000 | -0.105788 |

| | | | | |
|---|---|---|---|---|
| C | 1.206579 | -2.843522 | 0.0000 | -0.105788 |
| C | 0.000000 | 3.539419 | 0.0000 | -0.080890 |
| C | 0.000000 | -3.539419 | 0.0000 | -0.080890 |
| H | 3.391804 | 1.233846 | 0.0000 | 0.163583 |
| H | -3.391804 | -1.233846 | 0.0000 | 0.163583 |
| H | -3.391804 | 1.233846 | 0.0000 | 0.163583 |
| H | 3.391804 | -1.233846 | 0.0000 | 0.163583 |
| H | 2.146825 | 3.387117 | 0.0000 | 0.155641 |
| H | -2.146825 | -3.387117 | 0.0000 | 0.155641 |
| H | -2.146825 | 3.387117 | 0.0000 | 0.155641 |
| H | 2.146825 | -3.387117 | 0.0000 | 0.155641 |
| H | 0.000000 | 4.623680 | 0.0000 | 0.151532 |
| H | 0.000000 | -4.623680 | 0.0000 | 0.151532 |
